# Study on the impact of temperature, salts, sugars and pH on dilute solution properties of *Lepidium perfoliatum* seed gum

**DOI:** 10.1002/fsn3.2991

**Published:** 2022-07-22

**Authors:** Alireza Yousefi, Said Elmarhoum, Shahla Khodabakhshaghdam, Komla Ako, Ghader Hosseinzadeh

**Affiliations:** ^1^ Department of Chemical Engineering, Faculty of Engineering University of Bonab Bonab Iran; ^2^ CNRS, LRP Université Grenoble Alpes Grenoble France

**Keywords:** intrinsic viscosity, *Lepidium perfoliatum* seed gum, salt, sugar, surface activity, zeta potential

## Abstract

The functional properties of food gums are remarkably affected by the quality of solvent/cosolutes and temperature in a food system. In this work, for the first time, the chemical characterizations and dilute solution properties of *Lepidium perfoliatum* seed gum (LPSG), as an emerging carbohydrate polymer, were investigated. It was found that xylose (14.27%), galacturonic acid (10.70%), arabinose (9.07%) and galactose (8.80%) were the main monosaccharaide components in the LPSG samples. The uronic acid content of LPSG samples was obtained to be 14.83%. The average molecular weight and polydispersity index of LPSG were to be 2.34 × 10^5^ g/mol and 3.3, respectively. As the temperature was increased and the pH was decreased and the concentration of cosolutes (Na^+^, Ca^2+^, sucrose and lactose) presented in the LPSG solutions was enhanced, the intrinsic viscosity [*η*] and coil dimension (*R*_*coil*_, *V*_*coil*_, *υ*_*s*_) of LPSG molecular chains decreased. Activation energy and chain flexibility of LPSG were estimated to be 0.46 × 10^7^ J/kg.mol and 553.08 K, respectively. The relative stiffness parameter (*B*) of LPSG in the presence of Ca^2+^ (0.079) was more than that of Na^+^ (0.032). Incorporation of LPSG into deionized water (0.2%, w/v) diminished the surface activity from 76.75 mN/m to 75.70 mN/m. Zeta potential (*ζ*) values (−46.85 mV–−19.63 mV) demonstrated that dilute solutions of LPSG had strong anionic nature in the pH range of 3–11. The molecular conformation of LPSG was random coil in all the selected solution conditions. It can be concluded that temperature and presence of cosolutes can significantly influence on the LPSG properties in the dilute systems.

## Graphical abstract

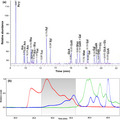

## 1 INTRODUCTION

By having various functional properties, hydrocolloids can be exploited for many applications in food and pharmaceutical industries. Nowadays, the hydrocolloids obtained from botanical resources have received much attention and have more acceptability by the consumers due to their bioactivity and biodegradability (Pourfarzad et al., 2021). *Lepidium perfoliatum* is a plant in the mustard family and it is native to Europe and Asia, and now distributed worldwide and broadly cultivated. The seeds of this plant have been consumed for a long time in traditional Iranian medical prescription owning to its pharmaceutical influences. It has been found that the mucilage obtained from the seeds of this plant, called *Lepidium perfoliatum* seed gum (LPSG), could be used as thickening and stabilizing agents due to shear thinning nature of its dispersions (Koocheki, Mortazavi, et al., 2009; Yousefi & Ako, 2020). The optimized procedure which leads to extract the LPSG form the seeds has been obtained by Koocheki, Taherian, et al. (2009), in which the extraction temperature of 48.1°C, pH of 8, water to seed ratio of 30:1 and process time of 1.5 h found to be the optimized conditions of extraction. Shear thinning behavior was observed for the LPSG dispersions at various temperatures (5–65°C) and concentrations (0.5%–2%, w/w), and also it was found that salts (NaCl, KCl, MgCl_2_, CaCl_2_) and pH (3–11) can remarkably influence the apparent viscosity of the dispersions (Koocheki et al., 2013).

Study on dynamic rheology of the LPSG dispersions (1.5%–3%, w/v) in the linear viscoelastic region has been shown that the dispersions exhibited viscoelastic properties at the given temperature of 5–85°C (Hesarinejad et al., 2014). Moreover, some functional properties of LPSG such as its emulsifying properties in the presence of whey protein concentrate (Soleimanpour et al., 2013), as well as its ability in fabrication of biodegradable nanocomposites have recently been proved (Seyedi et al., 2014).

Based on the literatures, study on the dilute solution properties of hydrocolloids gives a comprehensive insight into their fundamental characteristics. Finding out the molecular weight, the interaction with a solvent and the molecular shape, seems to be useful when the application of a particular hydrocolloid in a dilute solution and at a specific condition is desired (Hesarinejad et al., 2015). The surrounding medium of a macromolecules can affect the conformation of polyelectrolyte (Fathi et al., 2017), therefore a lot of studies have been found in which researchers focused on the effects of various solvent conditions on the conformation of hydrocolloids (Amini & Razavi, 2012; Behrouzian et al., 2014; Fathi et al., 2017; Hesarinejad et al., 2015; Mirabolhassani et al., 2016; Sherahi et al., 2018; Yousefi et al., 2014; Yousefi et al., 2016). In aqueous solution, the interaction of macromolecules and solvent's molecules can be changed by altering the quality of solvent through the use of commonly additives such as salts and sugars. Intrinsic viscosity, [*η*], is a criterion of the capability of macromolecules in solution to increment the viscosity of the solution. The intrinsic viscosity of a macromolecule in a solution is certainly associated with the quality of solvent. Accordingly, understanding any change in intrinsic viscosity can help to conceive the changes in molecular hydrodynamic volume, conformation and molecular associations (Amini & Razavi, 2012).

Analytical information such as chemical characteristics and structural properties are key factors to find the field of application and acceptability of an emerging hydrocolloid as an authorized food additives (Razavi et al., 2014). To the best of our knowledge, no study was taken up to investigate the chemical characterizations as well as the aqueous properties of LPSG at various conditions. Therefore, the main objectives of this work were (a) to characterize the chemical properties of LPSG powder using gas chromatography (GC), nuclear magnetic resonance (NMR) and gel permeation chromatography with multi‐angle light scattering detection (GPC/MALLS), and (b) to investigate the effect of varying temperatures (25–65°C) and different ions (Na^+^, Ca^2+^, 0–200 mM), sugars (sucrose 0%–40% w/v, and lactose 0%–10% w/v) on some hydrodynamic parameters in order to shed light on the behavior of LPSG in solution. As the salts and sugars used in this study are of the most abundant food additives, so study on the dilute solution of LPSG in the presence of these additives can also shed light on its behavior in real food systems.

## 2 MATERIAL AND METHODS

### 2.1 Materials

*Lepidium perfoliatum* seeds used in this study were provided locally from a medical plant supplier in Tabriz, Iran. The impurities of the seeds including dust, dirt, stones, chaff, immature and broken seeds were manually removed and the cleaned seed were subjected to extraction process. All chemicals used in this study were of analytical grade and supplied from Merck Company.

### 2.2 Extraction of *Lepidium perfoliatum* seed gum

For extraction of LPSG the method described by Koocheki, Taherian, et al. (2009) was implemented. In brief, the ratio of 30:1, distilled water: seed, at 48°C was prepared so that the seeds were completely soaked in the water. The pH of mixture solution was kept on 8 by adding adequate amounts of 0.1 M HCl and/or NaOH. The water‐seed mixture was manually stirred during 90 min of the extraction process. Afterwards, the swelled seeds were removed from the water the mucillages obtained were taken from the seeds and oven‐dried for 36 h at 50°C, and then milled and sieved by a sifter (100 mesh size).

### 2.3 Monosaccharide analysis

Monosaccharide composition of LPSG sample was determined according to the method of Kamerling et al. (1975) modified by Montreuil et al. (1986). The identification and determination of monosaccharides required hydrolysis by methanolysis of the polymer so as to get only monomers. The glycosidic residues were then trimethylsilylated in order to make them volatile. They were thus identified and determined by GC under form of O‐trimethylsilylated methylglycosides. Approximately 400 μg of lyophilized sample and 50 μg of pentaerythritol and 50 μg of myo‐inositol (internal references) were placed in a dry bath in the presence of 500 μl of a mixture of methanol/HCl 3 N (Supelco) for 4 h at 110°C. After cooling to room temperature, the methanolysate was neutralized with silver carbonate, and then 50 μl of acetic anhydride were added in order to reacetylate the osamines. After a night in the dark and at room temperature, the samples were centrifuged for 15 min at 3000 rpm and the supernatant was evaporated off under a nitrogen jet. The compounds were then dissolved in 70 μl of pyridine and incubated overnight at room temperature with 70 μl of sylon (BSTFA:TMCS, 99:1, Supelco). After gentle evaporation of the excess reagents under a nitrogen jet, the trimethylsilyl methylglycosides were taken up in 650 μl of dichloromethane and then injected by GC (AGILENT GC‐6850 system, in‐column injection, FID detector: flame ionization). The gas vector was hydrogen and the HP‐5MS type column (30 m, 0.25 mm internal diameter) was non‐polar. The temperature rise program was as follows: 120°C maintained for 1 min, then a gradient of 1.5°C/min up to 180°C followed by a gradient of 2°C/min up to 200°C.

### 2.4 Molecular weight measurement

Weight average molecular weight (*M*_*w*_) and number average molecular weight (*M*_*n*_) of LPSG were determined by GPC/MALLS. The samples were dissolved at a concentration of 1 mg/ml with the eluent used on the system. To improve the solubilization, it was necessary to pass the solutions to an ultrasonic bath and to heat at 35°C. Then, the samples were filtered through 0.2 μm before being injected onto two Shodex OHpak SB 806 M HQ columns placed in series at a temperature of 30°C. The system was calibrated by pullulan standard. The eluent used was 0.1 M sodium nitrate+0.03% sodium azide at a flow rate of 0.5 ml/min. The volume injected for each measurement was 100 μl. The elution time was 70 min, and the detection was performed using a Wyatt refractometer, a Shimadzu UV detector at the wavelength of 280 nm and a 3‐angle Wyatt light scattering detector. For this series of measurements, the value of the refractive index increment *dn*/*dc* was 0.155 ml/g. Polydispersity index (PDI) was also calculated (*PDI* = *M*_*w*_/*M*_*n*_). The analyses were carried out in triplicate.

### 2.5 ^1^H nuclear magnetic resonance analysis

To perform NMR analysis, about 10 mg LPSG powder have been dissolved in 1 ml D_2_O and ^1^H NMR spectra of the LPSG samples were carried out on a 400.13 MHz Bruker AVANCE III spectrometer. The proton spectrum was recorded at 353 K with a spectral width of 4006 Hz, 32,768 data points, 4.089 s of acquisition time, a relaxation time of 1 s and 64 scans.

### 2.6 Fourier‐transform infrared spectroscopy analysis

Infrared absorption spectrum of LPSG samples were recorded using IRAffinity‐1S spectrometer (Shimadzu) at a scan range of 4000 cm^−1^–400 cm^−1^ at a resolution of 2 cm^−1^. The LPSG samples were measured as a KBr pellet.

### 2.7 Zeta potential (*ζ*) analysis

Zeta potential of LPSG solutions (0.1%, w/v) was obtained in triplicates according to its electrophoretic mobility (*μ*) using dynamic light scattering technique (Zetasizer, Nano series, ZEN2600 model, Malvern Co.) at different pH of 3, 5, 7, 9, 11 at 25°C. For *kα* > > 1 (*k* is Debye—Hückel parameter and *α* is particle radius), the Smoluchowski equation was applied (Equation [1]):

(1) $$\zeta =\frac{\eta \mu }{\varepsilon }\;$$

here, *η* and *ε* are the viscosity and dielectric constant, respectively (Spatareanu et al., 2014).

### 2.8 Surface activity measurement

The surface activity of the LPSG solutions (0.1%–0.5%, w/v) was determined by measuring the surface tension at the air‐water interfaces using a tensiometer (GBX Digidrop). To calibrate the apparatus Milli‐Q water was used. At each concentration, three measurements were carried out at ambient temperature (25°C) and the average values were recorded.

### 2.9 Preparation of *Lepidium perfoliatum* seed gum solutions

By tray and assay experiments, LPSG solutions with concentration of 0.125% (w/v) were provided by the dissolution of 0.0625 g (d.b.) LPSG powder in 50 ml deionized water as well as salt (NaCl and CaCl_2_, 0–200 mM) and sugar (sucrose 0%–40% w/v, and lactose 0%–10% w/v) solutions at room temperature (300 rpm, 24 h). For the pH‐adjusted stock solution, pH of LPSG solutions was adjusted from 3 to 7 with 0.1 N HCl and 0.1 N NaOH under continuous stirring (300 rpm) at room temperature for 6 h. Then, the solutions were double filtered through 0.45 μm syringe filter with cellulose acetate membrane (Macherey–Nagel, Germany) to remove any insoluble particulate matter.

### 2.10 Intrinsic viscosity measurement

Dilute solutions of LPSG were prepared by adding a distinct amount of the solvents to the stock solution (0.125%, w/v). The intrinsic viscosity of all LPSG solutions were measured using an Ubbelohde capillary viscometer (Cannon Instruments Co.; viscometer constant, *k* = 0.007690 mm^2^s^−2^), immersed in a thermostatic water bath equipped with a precise temperature controller to maintain the selected temperatures (25, 35, 45, 55 and 65°C). The data reported for each dilute solution are the average of three replicates.

Based on the literature, various models have been used for calculation of intrinsic viscosity $\left[\eta \right]$, based on which the $\left[\eta \right]$ could be obtained from their intercepts (Huggins (Equation [2]) and {Kraemer (Equation [3])} or slopes {Higiro (Equation [4])}:

(2) $$\frac{\eta_{\mathrm{sp}}}{C}=\left[\eta \right]+k^{\prime {\left[\eta \right]}^{2}}C\;$$

(3) $$\frac{{\mathrm{ln\eta}}_{\mathrm{rel}}}{C}=\left[\eta \right]+k^{\prime \prime }{\left[\eta \right]}^{2}C$$

(4) $$\eta_{\mathrm{rel}}=\frac{1}{1+\left[\eta \right]C}$$

which in these equations, $\eta_{\mathrm{sp}}$ is specific viscosity, $\eta_{\mathrm{rel}}$ is relative viscosity, and *k′*, *k″* and *C* are, respectively, the Huggins constant, Kraemer constant and solute concentrations (g/dl).

### 2.11 Chain flexibility parameter

It is stated that in the Newtonian region of a polymer solution, increment in temperature leads to decrement in viscosity which follows Arrhenius equation (Equation [5]) (Stephen, 1995):

(5) η=AeEaRT

in which, *η* is the dynamic viscosity (Pa.s), *A* is a constant number, *E*_*a*_ is the activation energy of the flow process [kJ/(kg mol)], *R* is the universal gas constant (8.314 [kJ/(kg mol K)]) and *T* is the absolute temperature (*K*). The *E*_*a*_ value can be considered as a criterion of polymer chain flexibility. Accordingly, when the dynamic viscosity is replaced with the intrinsic viscosity, the slope obtained for natural logarithmic intrinsic viscosity against the inverse of absolute temperature (1/*T*) can be used for calculation of the chain flexibility of a polymer due to its relation to the *E*_*a*_.

### 2.12 Determination of relative stiffness parameter (*B*) and persistence length (*q*)

The stiffness parameter (*S*) of the LPSG chains was obtained based on the following equation from the intrinsic viscosity's slope at various ionic strengths versus the inverse square root of ionic strength (*I* ^−0.5^) plot (Lai & Chiang, 2002):

(6) $$\left[\eta \right]={\left[\eta \right]}_{\infty }+SI^{-0.5}$$

in which, [*η*]_*∞*_ exhibits the intrinsic viscosity at infinite ionic strength. As the constant *S* is powerfully molecular weight dependent, therefore *B* parameter has been introduced as an independent stiffness parameter which can be calculated as follows:

(7) $$S=B{\left({\left[\eta \right]}_{0.1}\right)}^{\nu }$$

in this equation, the *ν* parameter was found to be within the range of 1.2–1.4, so the mean value of 1.3 is often used as a constant number. Also, the value of [*η*]_0.1_ indicates the intrinsic viscosity at an ionic strength of 0.1 M. In this case, there is another character so‐called “persistence length” (*q*) which is a criterion of the length and it is related to *B* parameter (Eteshola et al., 1996). In brief, *q* is a measure of the length over which the chain ″persists″ in the direction of the first bond of the chain. The equation which shows the relation between *B* and *q* parameters is as follows:

(8) $$q=\frac{0.26}{B}$$

### 2.13 Estimation of the molecular conformation

For this reason, the exponent *b* from the slope of a double logarithmic plot of specific viscosity against concentration was calculated. It is stated that the conformation of polysaccharides can be estimated through this parameter (Higiro et al., 2007):

(9) $$\eta_{\mathrm{sp}}=aC^{b}$$

### 2.14 Shape and swollen volume parameters

The intrinsic viscosity is associated with shape function and swollen specific volume, which are two meaningful molecular parameters and can be obtained based on the following equation (Equation [10]):

(10) $$\left[\eta \right]=\upsilon .\upsilon_{s}$$

here, *υ* parameter is the shape function, which also known as the viscosity increment, and *υ*_*s*_ parameter is swollen specific volume or voluminosity (Antoniou et al., 2010). It is reported that the *υ* parameter is corresponded to an anhydrous macromolecule will essentially expand when suspended or dissolved in solution due to the solvent association. Moreover, the *υ*_*s*_ parameter can be considered as a criterion of the solvent associated with the macromolecule or by definition, it is the volume of macromolecule in solution per its unit anhydrous mass (Mirabolhassani et al., 2016).

The swollen specific volume is associated with the relative viscosity which it can be obtained through the intercept of a plot of the following equation (Equation [11]) versus concentration (Yousefi et al., 2014):

(11) $$Y=\frac{\eta_{\mathrm{rel}}^{0.5}-1}{C\left(1.35\eta_{\mathrm{rel}}^{0.5}-0.1\right)}$$

so, the *υ* parameter can be attained from specific swollen volume and intrinsic viscosity using Equation (11).

### 2.15 Coil radius and volume

From the Einstein viscosity relation, Antoniou et al. (2010) have stated that the hydrodynamic radius (*R*_*coil*_) can be determined using the following equation:

(12) $$R_{\text{coil}}={\left[\frac{3\left[\eta \right]M_{w}}{10\pi N_{A}}\right]}^{0.33}$$

in which, *M*_*w*_ and *N*_*A*_ are, respectively, the average molecular weight and the Avogadro's number (6.022 × 10^23^ mol^−1^).

By the assumption that we have a sphere‐like coil, therefore the corresponding coil volume (*V*_*coil*_) can be calculated from the following equation:

(13) $$V_{\text{coil}}=\frac{4}{3}\pi R_{\text{coil}}^{3}$$

### 2.16 Statistical analysis

In this study, SPSS 17 software (SPSS Inc.) was used for statistical analyses of the results obtained from dilute solutions of LPSG at different conditions (temperatures, salt and sugar concentrations). The results were evaluated with 5% significance level (*p* < .05) by one‐way analysis of variance (anova), and the mean values were compared using Duncan test. Data were obtained at least in duplicate and presented as the mean ± standard deviation.

## 3 RESULTS AND DISCUSSION

### 3.1 Monosaccharide content of *Lepidium perfoliatum* seed gum

**FIGURE 1:**
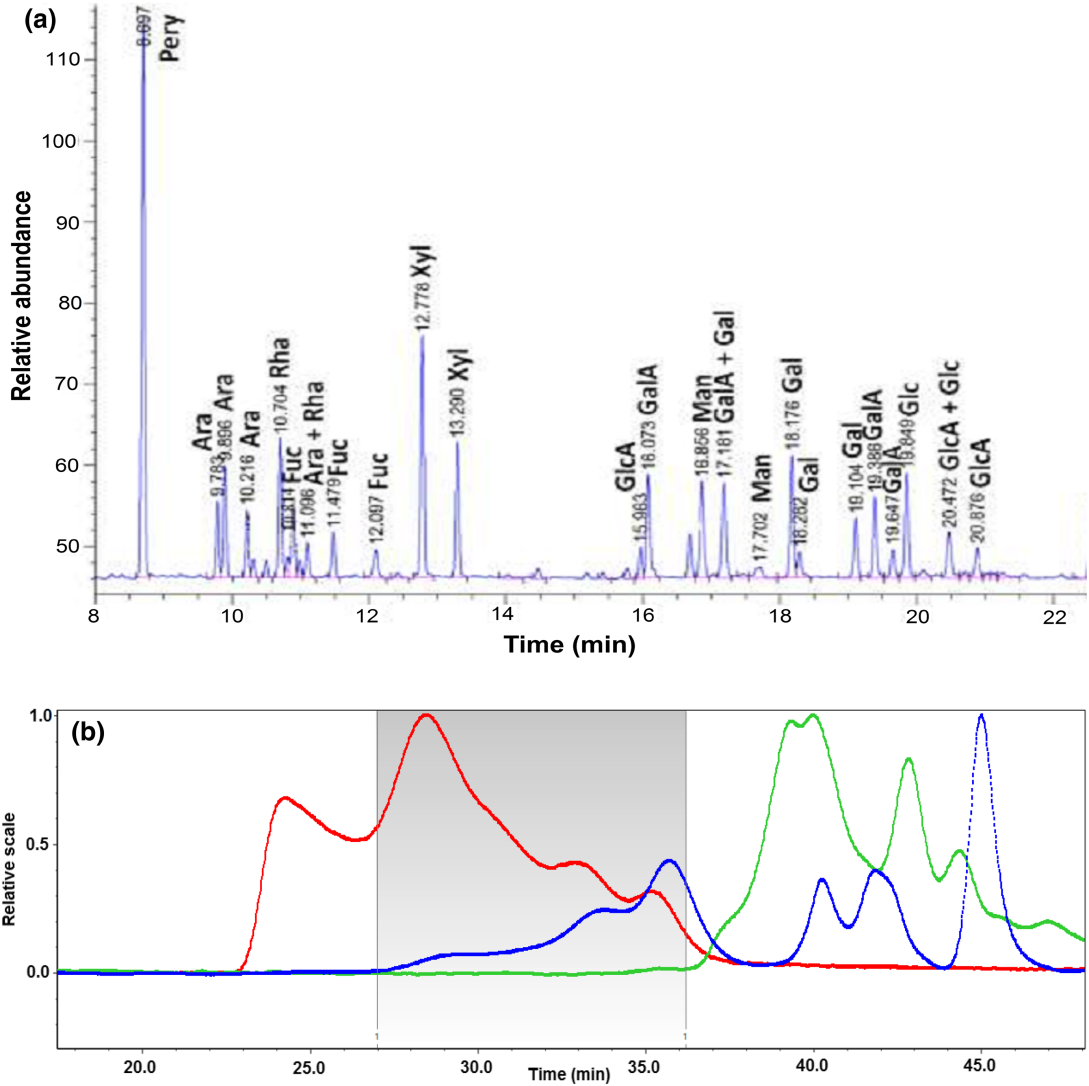
(a) Chromatogram obtained after the analysis of trimethylsilyl derivatives of LPSG by GC–MS; (b) GPC/MALLS chromatogram obtained for LPSG sample. The red line chromatogram corresponds to light scattering detection, the blue line chromatogram corresponds to refractometric detection, and the green line chromatogram corresponds to UV detection at a wavelength of 280 nm. The integral range of GPC was 27–37 min (the gray window in the graph); LPSG, *Lepidium perfoliatum* seed gum

The chromatogram obtained after analysis of trimethylsilyl derivatives of the LPSG sample by GC is shown in Figure 1a. The results of the analysis of monosaccharides for LPSG powder is also exhibited in Table 1. As it can be seen, carbohydrates are the major component of LPSG (66.02%). This amount shows the same level of LPSG total carbohydrate for LPSG compared to some hydrocolloids such as sage seed gum (69.96%–71.05%) (Razavi et al., 2014), guar gum (71.1%) (Busch et al., 2015), but was lower than that of cress seed gum (87.4%) (Karazhiyan et al., 2011), *Prosopis ruscifolia* seed gum (76%) (Busch et al., 2015) and *Opuntia ficus indica* gum (88.85%) (Salehi et al., 2019). It was found that xylose (14.27%), galacturonic acid (10.70%), arabinose (9.07%) and galactose (8.80%) were the main, while fructose (3.50%), mannose (4.02%) and glucuronic acid (4.13%) were the minor monosaccharaide components in the LPSG samples.

**TABLE 1:** Monosaccharide composition, weight average molecular weight (*M*_*w*_), number average molecular weight (*M*_*n*_) and polydispersity index (PDI) values of LPSG

Composition	Amount (%)
Fructose	3.50 ± 0.28
Arabinose	9.07 ± 0.45
Galactose (G)	8.80 ± 0.48
Glucose	5.42 ± 0.48
Xylose	14.27 ± 0.66
Mannose (M)	4.02 ± 0.26
Rhamnose	6.41 ± 0.29
Glucuronic acid (GLUA)	4.13 ± 0.59
Galacturonic acid (GALA)	10.70 ± 0.56
Total carbohydrate	66.02 ± 2.66
Uronic acids (GLUA+GALA)	14.83 ± 0.57
G/M	218.91
Galactomanan (G + M)/total carbohydrate	19.42
Characteristic	Amount
Weight average molecular weight (*M*_*w*_)	234.1 ± 0.3 (kDa)
Average molecular weight (*M*_*n*_)	70.3 ± 0.8 (kDa)
The polydispersity index (*PDI*)	3.33 ± 0.04

Abbreviation: LPSG, *Lepidium perfoliatum* seed gum.

High content of uronic acid attained for LPSG samples (14.83%) proves the polyelectrolyte nature of the gum. This extent was comparably the same with that for psyllium gum (15.9%) and cress seed gum (15%) but higher than that for *Alyssum homolocarpum* seed gum (5.63%), gum ghatti (12.83%), and also lower than that for flaxseed gum (21.0%–25.1%), xanthan (21.9%), sage seed gum (28.2%–32.2%), *Cissampelos pareira* pectin (70.56%) (Hesarinejad et al., 2015; Razavi et al., 2014).

The ratio of galactose to mannose (G/M) for the LPSG samples (2.19) was close to that of sage (1.93), *Gleditsia melanacantha* (2.30) seed gums, but higher than that for fenugreek (1.2), *Adenanthera pavonina* (1.35), and mesquite (1.1–1.50) seed gums, and also lower than that for Cassia (5.0) and cress (8.2) seed gums (Karazhiyan et al., 2011; Razavi et al., 2014). According to the monosaccharide compositions of LPSG and low content of galactomannan (19.42%) (Table 1), it can be deduced that it has a different structure in comparison with the other gums like xanthan, guar and sage seed. In fact, LPSG is neither galactommanan nor glucomannan, but may have the structure of galactan‐type polysaccharides with a highly branched xyloarabinan. Similar results have been reported for *Opuntia ficus‐indica* mucilage (Di Lorenzo et al., 2017).

### 3.2 Molecular weight of *Lepidium perfoliatum* seed gum

Molecular weight of Carbohydrate Polymers (*M*_*w*_ *and M*_*n*_) as well as PDI remarkably influences their physicochemical and functional attributes. Based on the results obtained from the GPC profile of the LPSG sample (Figure 1b), the values of *M*_*w*_ and *M*_*n*_ and the resultant PDI values were attained which are tabulated in Table 1. The value of *M*_*w*_ for the LPSG samples was found to be 2.34 × 10^5^ Da, which is close to that of *Alyssum homolocarpum* seed gum (3.66 × 10^5^ Da) (Hesarinejad et al., 2015) and sage seed gum (4.25 × 10^5^ Da) (Razavi et al., 2014). On the contrary, this value was much lower than those reported for guar gum (2.07 × 10^6^ Da) (Busch et al., 2015), xanthan gum (4.05 × 10^6^ Da) (Viturawong et al., 2008), Balangu seed gum (3.60 × 10^6^ Da) (Amini & Razavi, 2012), *Descurainia Sophia* seed gum (2.1 × 10^6^ Da) (Sherahi et al., 2018) and *Opuntia ficus indica* fruit gum (3.67 × 10^6^ Da) (Salehi et al., 2019). Based on the literature, such low molecular weight polymers can potentially be used as emulsifier, plasticizer, drug deliver, dispersants and crystal growth modifiers (Soleimanpour et al., 2013).

Homogeneity and molecular weight distribution of polymers are defined by the PDI. This high value of PDI observed for the LPSG samples indicates large dispersity of molecular weight of its fractions. We know that the *M*_*n*_ is biased toward the lower molecular‐weight fraction and the *M*_*w*_ is biased toward the higher molecular‐weight fraction. Consequently, a high value of PDI is an indication of presence of a large number of low‐molecular‐weight molecules together with a small number of very large molecules.

### 3.3 ^1^H nuclear magnetic resonance and FTIR analyses

**FIGURE 2:**
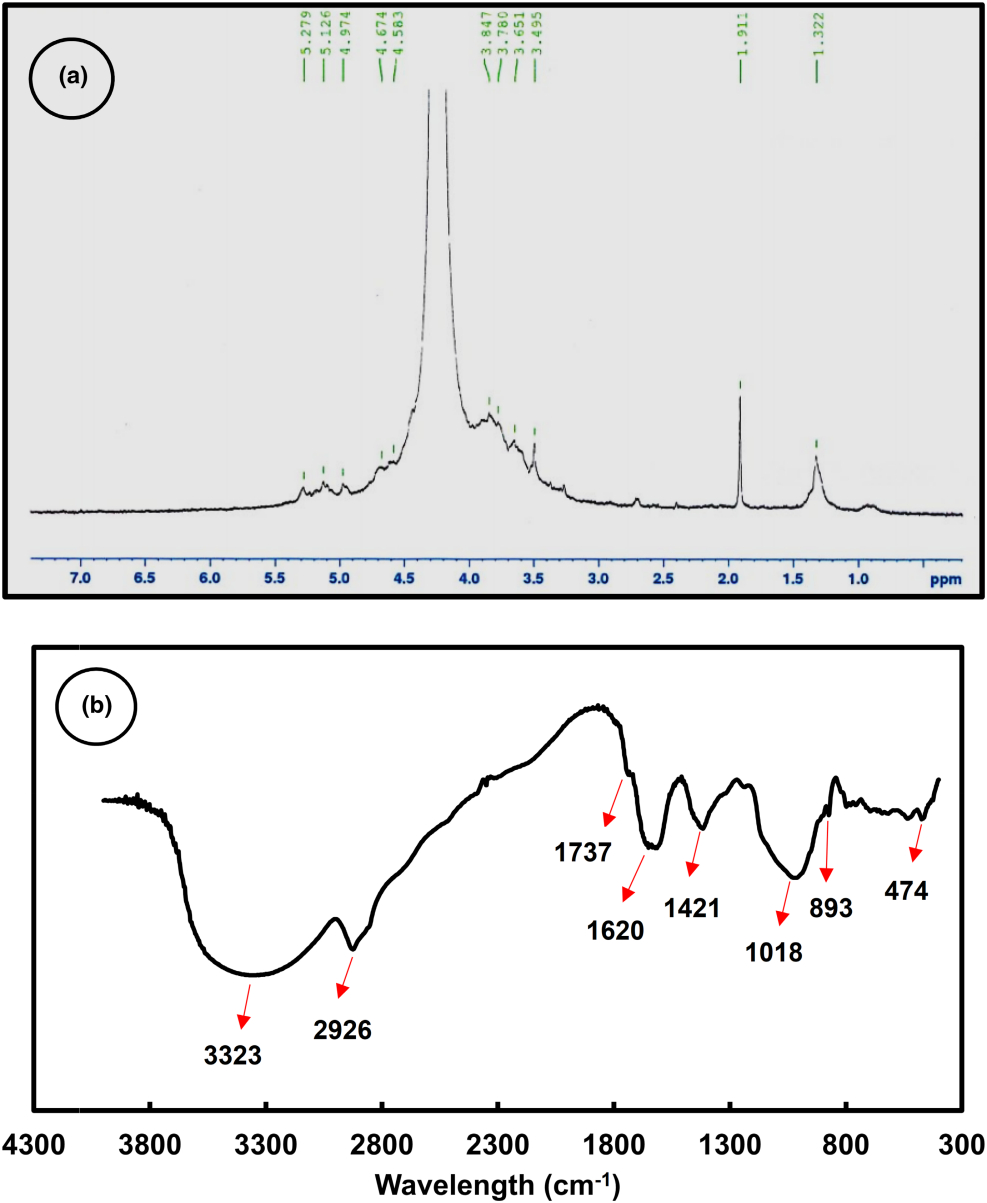
(a) ^1^H nuclear magentic resonance spectrum of LPSG; (b) FTIR spectrum of LPSG; LPSG, *Lepidium perfoliatum* seed gum

^1^H NMR spectrum of LPSG was recorded which is depicted in Figure 2a. As it can be seen, the diagnostic resonances were in the range of 1.32–5.28 ppm. The signals within the range of 3.1–4.3 ppm are assigned to non‐anomeric ^1^H (H_2_–H_6_), whereas the range 4.3–4.9 and 4.9–5.5 ppm are, respectively, associated to α‐anomeric and β‐anomeric ^1^H (Whistder & BeMiller, 1959). The signals obtained between 3.1 to 3.8 ppm have also been assigned to *–O–CH*_*3*_ residue (Nep & Conway, 2010), indicating the presence of methylated (*–CH*_*3*_) monosaccharide in the samples. This result was in agreement with that of obtained from GC–MS analysis which showed the presence of rhamnose (6.41%) in the monosaccharide compositions (Table 1). The resonances observed at 1.32 and 1.91 ppm are, respectively, associated with the existence of ether group (*CH*_*3*_ *–CH*_*2*_ *–O*) and O‐acetylated galacturonic acid residues in the LPSG structure (Wang et al., 2017).

The FTIR spectrum of LPSG is depicted in Figure 2b. Polysaccharide's fingerprint region can be observed at the bands between 900 to 1200 cm^−1^, and the bound at 893 cm^−1^ of this region is corresponded to α and β linkages in the polymer structure (Jin, 2012). The observed bands around 1421 and 1620 are related to the presence of symmetric stretching vibration of COO– in LPSG structure. The stretching vibration of C – H in a methylene group (CH_2_) and/or double overlapping with OH groups is arisen at 2926 cm^−1^. The absorption bands in the range of 3100 to 3500 indicate the presence of –OH groups. Sharma and Mazumdar (2013) have stated that hydrogen bonding involving the hydroxyl groups of glucopyranose rings causes the mentioned region.

### 3.4 Zeta potential (*ζ*)

**FIGURE 3:**
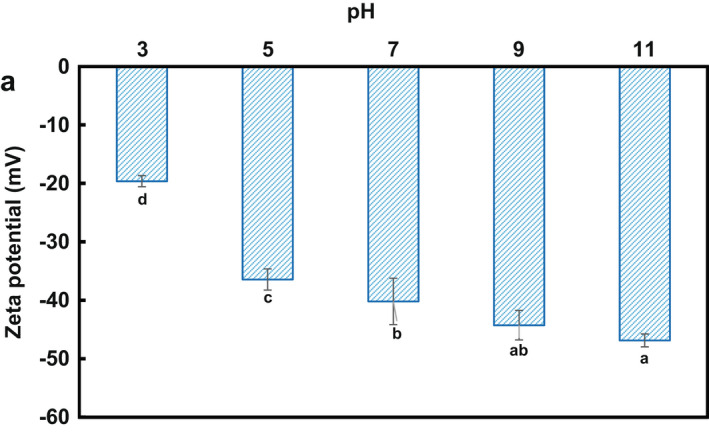
Zeta potential (*ζ*) of LPSG at different pH. Values with different letters are significantly different (*p* < .05); LPSG, *Lepidium perfoliatum* seed gum

The type and amount of electrostatic interactions for charged biopolymers can be implied by the zeta potential measurement. The results obtained for the zeta potential of LPSG solutions indicated a strong anionic nature (*ζ* = − 46.85 mV) at alkaline pH of 11; however, the extent of negativity for this characteristic was gradually diminished with decreasing pH and reached to −19.63 mV at pH of 3 (Figure 3). This anionic nature probably comes from high content of acidic sugars (glucrunic acid and galactrunic acid, ≈ 23% of total monosaccharides (Table 1)), which are partially ionized in water and lead to negative charge functional group (–COO^−^) in the solutions (Timilsena et al., 2016). The value of zeta potential gained in this study (at neutral pH, −40.18 mV) was similar to that for gum arabic (−33.0–−44.3 mV) (Li et al., 2019), but higher than that of the hydrolyzed peach gum polysaccharide (≈ −35 mV) (Huang & Zhou, 2014) and *Alyssum homolocarpum* seed gum (−25.81 mV) (Hesarinejad et al., 2015). The electrical conductivity of the LPSG solutions was found to be decreased from 368 to 161 μScm^−1^ with increasing pH from 3 to 11 (data not shown). The electrical conductivity characteristic has been found to be related to the interaction of water molecules with the functional groups (–OH and –COO^−^) in the side chains of the biopolymers (Morales‐Sánchez et al., 2015).

### 3.5 Surface activity

**FIGURE 4:**
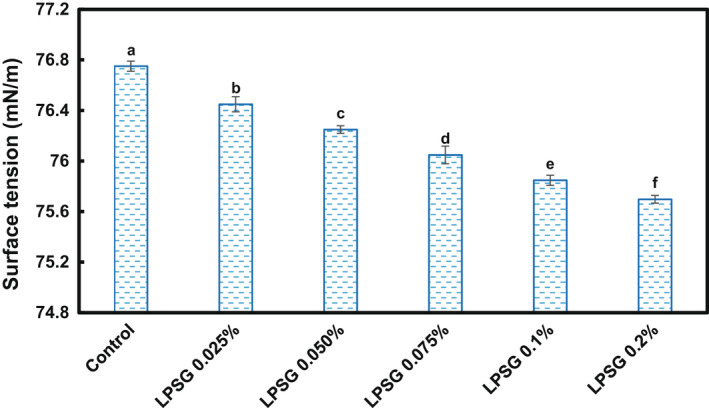
Surface tension of LPSG solutions as a function of concentration. Values with different letters are significantly different (*p* < .05); LPSG, *Lepidium perfoliatum* seed gum

The surface tension of LPSG solutions in air‐water interface as a function of concentration is depicted in Figure 4. As the concentration of LPSG was elevated from 0% to 0.2%, the surface tension value gradually decreased from 76.75 mN/m to 75.70 mN/m. It has been stated that increase in surface tension as a results of increase in polymer concentration could be attributed to gelation and high viscosity (Naji‐Tabasi et al., 2016). In dilute solution domain, it can be supposed that at higher concentration, larger amounts of macromolecules can migrate to the air‐water interface, and consequently, more reduction in surface tension occurs. Furthermore, it has also been mentioned that the surface tension of gums is attributed to existence of protein in their compositions (Garti et al., 1997). Accordingly, the presence of 4.6% protein in the composition of LPSG powder (Yousefi & Ako, 2020) is expected to contribute to surface activity of its dispersions. More reduction in surface activity of water has been reported affected by the solutions of Australian chia seed gums (Timilsena et al., 2016). Hydrocolloid gums exhibit low degree of surface activity due to their hydrophilic nature. It is reported that gum Arabic and xanthan gum at 10 g/kg diminish the surface tension of water to 60 mN/m and 42 mN/m, respectively (Timilsena et al., 2016).

### 3.6 Intrinsic viscosity

The hydrodynamic volume of macromolecules which is regarded to the dimension of polymer chain can be sensed by the intrinsic viscosity. The values of intrinsic viscosity of LPSG in the selected conditions calculated from the equations of 2–4 are tabulated in Table 2. A good fitness results was observed for Huggins and Kraemer models (.83 ≤ *R* ^2^ ≤ .99) in which the [*η*] is, respectively, calculated by extrapolation of *η*_*sp*_ */C* and *ln*_*rel*_ */C* values to the zero concentration. Many studies have revealed that the [*η*] can be calculated much precisely by the models in which this parameter presents in their slopes. Among the models, Higiro one (Equation (4)) showed the best fitting result to predict the [*η*] in all conditions examined (.97 ≤ *R* ^*2*^ ≤ .99). As a result, the resulting [*η*] value of this model was considered for investigation of the influence of the selected conditions on aqueous solution properties of LPSG. The intrinsic viscosity of LPSG (25°C, distilled water) has been compared with that of some other hydrocolloids, which indicates that LPSG has a medium intrinsic viscosity (Table 3). As seen, this may be attributed to the low molecular weight of LPSG (234.1 kDa) compared to many other hydrocolloids.

**TABLE 2:** Values of intrinsic viscosity, exponent *b* and berry number for LPSG at the selected solution conditions

Solution conditions		Huggins		Kraemer		Higiro		*b* parameter	Berry number *C*[*η*]
		[*η*] (dl/g)	*R* ^2^	[*η*] (dl/g)	*R* ^2^	[*η*] (dl/g)	*R* ^2^		
Temperature (°C)	25	7.87 ± 0.19^a^	.97	7.91 ± 0.26^a^	.93	13.53 ± 0.33^a^	.98	1.12 ± 0.02^a^	0.42–0.79
	35	6.44 ± 0.22^b^	.94	6.67 ± 0.18^b^	.97	12.98 ± 0.24^b^	.99	1.11 ± 0.02^ab^	0.40–0.77
	45	5.91 ± 0.13^c^	.94	5.80 ± 0.09^c^	.95	12.04 ± 0.25^c^	.99	1.10 ± 0.01^b^	0.38–0.74
	55	5.10 ± 0.21^d^	.96	4.42 ± 0.23^d^	.91	11.57 ± 0.09^d^	.98	1.10 ± 0.00^b^	0.36–0.69
	65	4.74 ± 0.14^e^	.98	4.09 ± 0.04^e^	.94	10.89 ± 0.13^e^	.98	1.08 ± 0.00^c^	0.34–0.61
NaCl (mM)	10	3.51 ± 0.26^a^	.94	3.37 ± 0.11^a^	.86	7.41 ± 0.08^a^	.97	1.14 ± 0.00^c^	0.16–0.36
	50	2.18 ± 0.09^b^	.89	2.40 ± 0.10^b^	.92	5.19 ± 0.08^b^	.98	1.17 ± 0.02^b^	0.13–0.26
	100	1.63 ± 0.09^c^	.90	1.72 ± 0.05^c^	.92	4.60 ± 0.12^c^	.97	1.19 ± 0.00^b^	0.12–0.23
	200	1.46 ± 0.04^d^	.96	1.66 ± 0.07^c^	.95	3.88 ± 0.06^d^	.98	1.21 ± 0.01^a^	0.10–0.21
CaCl_2_ (mM)	10	2.48 ± 0.16^a^	.95	2.83 ± 0.15^a^	.91	5.34 ± 0.07^a^	.99	1.16 ± 0.02^c^	0.16–0.29
	50	1.77 ± 0.10^b^	.99	2.01 ± 0.08^b^	.83	3.46 ± 0.10^b^	.98	1.18 ± 0.01^c^	0.14–0.26
	100	1.30 ± 0.03^c^	.91	1.48 ± 0.03^c^	.90	2.64 ± 0.04^c^	.98	1.23 ± 0.02^b^	0.12–0.28
	200	1.16 ± 0.03^d^	.98	1.29 ± 0.06^d^	.89	2.11 ± 0.05^d^	.99	1.28 ± 0.03^a^	0.11–0.23
Sucrose (%)	10	5.22 ± 0.11^a^	.99	5.64 ± 0.13^a^	.94	10.07 ± 0.15^a^	.98	1.06 ± 0.00^c^	0.22–0.50
	20	4.71 ± 0.17^b^	.92	4.55 ± 0.19^b^	.94	8.69 ± 0.21^b^	.98	1.09 ± 0.02^b^	0.20–0.46
	30	3.45 ± 0.12^c^	.88	3.38 ± 0.14^c^	.97	6.76 ± 0.13^c^	.99	1.11 ± 0.02^ab^	0.16–0.37
	40	2.06 ± 0.08^d^	.86	2.17 ± 0.05^d^	.96	6.53 ± 0.03^d^	.97	1.13 ± 0.01^a^	0.13–0.29
Lactose (%)	2.5	6.11 ± 0.18^a^	.93	6.32 ± 0.16^a^	.93	11.90 ± 0.27^a^	.97	1.17 ± 0.03^a^	0.30–0.69
	5	5.60 ± 0.15^b^	.97	5.43 ± 0.09^b^	.94	9.94 ± 0.18^b^	.99	1.09 ± 0.01^b^	0.25–0.56
	7.5	4.28 ± 0.23^c^	.97	4.70 ± 0.20^c^	.88	8.57 ± 0.19^c^	.97	1.08 ± 0.02^bc^	0.20–0.46
	10	3.79 ± 0.07^d^	.90	3.98 ± 0.11^d^	.91	8.01 ± 0.11^d^	.98	1.06 ± 0.01^c^	0.19–0.43
pH	3	5.19 ± 0.06^c^	.94	5.35 ± 0.13^c^	.90	11.19 ± 0.16^c^	.98	1.14 ± 0.02^a^	0.27–0.56
	5	6.51 ± 0.10^b^	.89	6.89 ± 0.08^b^	.92	13.06 ± 0.09^b^	.97	1.11 ± 0.00^b^	0.33–0.65
	7	8.04 ± 0.07^a^	.93	8.11 ± 0.19^a^	.90	13.84 ± 0.21^a^	.97	1.07 ± 0.01^c^	0.41–0.74

Abbreviation: LPSG, *Lepidium perfoliatum* seed gum.

^a–d^ For each solution condition, values in columns with different letters are significantly different (*p* < .05).

**TABLE 3:** Comparison of the average molecular weight and intrinsic viscosity of LPSG with some hydrocolloids

Hydrocolloid	*M*_*w*_ (kDa)	Temperature (°C)/solvent	[*η*] (dl/g)	Reference
LPSG	234.1	25/water	13.53	Current study
Xanthan	4050	25/water	110.34	Viturawong et al. (2008)
Locust bean gum	2080	25/water	14.20	Wu et al. (2009)
Guar Gum	2910	25/water	15.80	Wu et al. (2009)
κ‐Carrageenan	‐	25/water	41.20	Nickerson et al. (2004)
Dextran	500	20/water	0.49	Antoniou et al. (2010)
Tara gum	2230	25/water	14.55	Wu et al. (2009)
Pectin (high‐methoxy)	156	20/0.1 M phosphate buffer	4.06	Morris et al. (2002)
Pectin (low‐methoxy)	‐	25/water	24.50	Sato et al. (2008)
Fenugreek gum	3230	25/water	15.10	Wu et al. (2009)
Konjac glucomannan	210–740	20/0.1 M phosphate buffer	4.75–13	Kök et al. (2009)
Balangu seed gum	3650	20/water	72.36	Amini and Razavi (2012)
Sage seed gum	1500	25/water	7.59	Yousefi et al. (2014)
Cress seed gum	‐	25/water	3.92	Behrouzian et al. (2014)
*Alyssum homolocarpum* seed gum	336	25/water	18.34	Hesarinejad et al. (2015)
Basil seed gum	1730	25/water	11.38	Mirabolhassani et al. (2016)
*Prunus armeniaca* gum	‐	25/water	3.06	Fathi et al. (2017)
*Descurainia sophia* seed gum	‐	25/water	8.73	Sherahi et al. (2018)

Abbreviation: LPSG, *Lepidium perfoliatum* seed gum.

From 25°C, for every 10°C increment in solution temperature, the [*η*] of LPSG was slightly decreased, which was about 4%, 7%, 4% and 6%, respectively. This effect may be in connection with an abrupt change in the gyration of LPSG macromolecules caused by increasing chain flexibility and/or molecular contraction. Moreover, lower temperature is favored for polymer‐solvent interaction, giving negative enthalpy of polymer‐solvent mixing (Kasaai, 2008).

From the results at 25°C, in the presence of cations (Na^+^ and Ca^2+^), a large decrease in intrinsic viscosity of LPSG was observed (45%–71% for Na^+^, and 61%–84% for Ca^2+^), which was more pronounced at higher ionic concentrations. Furthermore, more remarkable reduction influence of di‐cation Ca^2+^ on intrinsic viscosity of LPSG was obtained in comparison with mono‐cation Na^+^, indicating Na^+^ was less effective in decreasing molecular dimensions of LPSG than Ca^2+^. The same behavior has been reported for Balangu seed gum (Amini & Razavi, 2012), sage seed gum (Yousefi et al., 2014) and cress seed gum (Behrouzian et al., 2014). This decrease is due to the charge screening of electrostatic repulsions of the tri‐saccharide side‐chain, which leads a more compact conformation and causes a reduction in hydrodynamic size of the molecule (intrinsic viscosity). In addition, the impacts of ions on intrinsic viscosity may be related to the ionic nature of some macromolecules, and since LPSG solution is a polyelectrolytes (with zeta potential of −40.18 mV and uronic acid content of 14.83%), so its hydrodynamic characteristics will be influenced by the presence of ions in the solution (Sherahi et al., 2018).

As the concentration of sucrose (from 10% to 40%) and lactose (from 2.5% to 10%) increased, the extent of [*η*] was decreased 35% and 33%, respectively. In proportion to the selected concentrations, this result implies more reduction in intrinsic viscosity of LPSG in the presence of lactose compared to sucrose. Similar results have been reported for other emerging carbohydrate polymers such as Balangu seed gum (Amini & Razavi, 2012) and basil seed gum (Mirabolhassani et al., 2016). The competition between the sugars and LPSG molecules for water is probably the reason for consequent diminution of intrinsic viscosity with increasing the sugars concentration (Behrouzian et al., 2014). On the other hands, it can be inferred that as the concentration of sugars increases, the probability of the interaction of –OH groups of LPSG molecules with –C–OH groups of sugars enhances compared to their interaction with –OH groups of water. It is clear that the less the interaction of water‐LPSG molecules, the more the intramolecular associations and decrement in intrinsic viscosity. It was found that a decrease in pH from 7 to 3, resulted in a decrease in intrinsic viscosity of LPSG solutions from 13.84 to 11.19 (dl/g). It has been reported that this diminution of viscosity under strong acidic condition is related with the decrease of molecular weight, resulting in the shorter length of side chain of macromolecules (Bak & Yoo, 2018). The same results were reported for the impact of acidic conditions on the apparent viscosity of LPSG dispersions at the pH range of 3–5 (Koocheki et al., 2013).

### 3.7 Chain flexibility and activation energy

**FIGURE 5:**
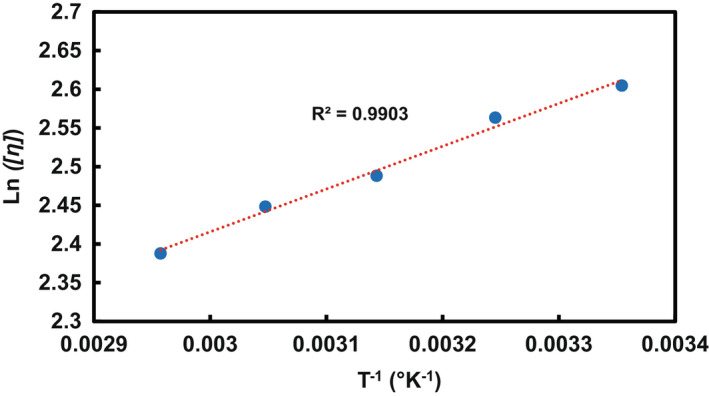
An Arrhenius‐type plot for determination of chain flexibility parameter of LPSG in deionized water; LPSG, *Lepidium perfoliatum* seed gum

The slope of the curve obtained by the plot of *ln*[*η*] against the reciprocal of absolute temperature (1*/T*) gives a criterion of molecular chain flexibility (Figure 5). Accordingly, the value of 553.08 K was calculated as the chain flexibility factor (*E*_*a*_ */R*) for LPSG at the temperature within the range of 25–65°C. This value was close to the value of 618.54 K reported for *Alyssum homolocarpum* seed gum (Hesarinejad et al., 2015) and 665.35 K for basil seed gum (Mirabolhassani et al., 2016), but was lower than that of several hydrocolloids such as 1100 K for xanthan (Milas & Rinaudo, 1986), 1156.53 K for Balangu seed gum (Amini & Razavi, 2012), 3046.45 K for sage seed gum (Yousefi et al., 2014) and 1353 K for *Descurainia Sophia* seed gum (Sherahi et al., 2018). Accordingly, it can be proposed that LPSG has exactly low level of flexibility. This characteristic comes from the low value of *M*_*w*_ of LPSG (234.1 kDa, Table 1), and it is stated that low molecular weight macromolecules cannot be able to entangle sufficiently in solutions and have lower degree of flexibility (Chen & Tsaih, 1998). The molecules with lower flexibility, due to lower degree of entanglement and stronger structure, could be exploited in encapsulation process for holding the liquid inside the capsule (Chen et al., 1996). So, LPSG can be an appropriate encapsulator in food and pharmaceutical systems.

Generally, the value of activation energy (*E*_*a*_) for most of Carbohydrate Polymers is in the range of 1 × 10^7^ to 1 × 10^8^ [J/(kg mol)] (Chen & Tsaih, 1998). The value of 0.46 × 10^7^ [J/(kg mol)] determined for LPSG was comparable to the values of 0.51 × 10^7^, 0.55 × 10^7^ and 0.83 × 10^7^ [J/(kg mol)] obtained, respectively, for *Alyssum homolocarpum* seed gum, basil seed gum and *Prunus armeniaca* gum (Fathi et al., 2017; Hesarinejad et al., 2015; Mirabolhassani et al., 2016); however, it was much lower than those reported for chitosan {2.005 × 10^7^ (J/[kg mol])} and sage seed gum {2.53 × 10^7^ (J/[kg mol])} (Chen & Lin, 1992; Yousefi et al., 2014). This value indicates low temperature sensitivity of LPSG compared to the most of hydrocolloids.

### 3.8 Relative stiffness parameter (*B*) and persistence length (*q*)

Hydrocolloids mostly are utilized in the food/pharmaceutical systems which commonly contain some salts, therefore it is important to find out the impact of ions on their rheological properties (Yousefi et al., 2014). Based on the Equations (6) and (7), the stiffness (*S*), the relative stiffness (*B*) and the intrinsic viscosity at infinite ionic strength ([*η*]_*∞*_) parameters were calculated in the presence of the selected cations (*ν* was supposed to be 1.3) (Table 4). As expected, a greater value of ([*η*]_*∞*_), accompanied with lower values of *S* and *B* were gained in the presence of Na^+^ compared to Ca^2+^, implying again more flexibility of LPSG molecules in monovalent salt solutions. A greater extent of contraction of LPSG molecules in the presence of Ca^2+^ has previously been proved by the results of intrinsic viscosity (Table 2). Consequently, it can be concluded that LPSG molecule in confronting with monovalent cations has a rather flexible conformation, whilst in the presence of divalent ones has a semi‐flexible/stiff conformation. In comparison, the *B* values of LPSG in the presence of Na^+^ (0.032) and Ca^2+^ (0.079) were found to be lower than those reported for Balangu seed gum (Amini & Razavi, 2012), sage seed gum (Yousefi et al., 2014), *Prunus armeniaca* gum (Fathi et al., 2017) and *Descurainia Sophia* seed gum (Sherahi et al., 2018), indicating stiffer conformation for LPSG in comparison to the mentioned hydrocolloids.

**TABLE 4:** The molecular parameters estimated for LPSG by using the equations (6–8)^ab^

Solvent	[*η*_*∞*_] (dl/g)	*S*	*B*	*q* (nm)
NaCl	3.105 ± 0.089^a^	0.436 ± 0.012^b^	0.032 ± 0.003^b^	8.125 ± 1.087^a^
CaCl_2_	1.385 ± 0.024^b^	0.698 ± 0.019^a^	0.079 ± 0.005^a^	3.291 ± 0.296^b^

Abbreviation: LPSG, *Lepidium perfoliatum* seed gum.

^a,b^ Values in columns with different letters are significantly different (*p* < .05).

The persistence length parameter (*q*) of LPSG in the selected ionic solutions calculated using the Equation (8) is represented in Table 4. As it can be seen, the *q* values for LPSG in the presence of Na^+^ (8.125 nm) was greater than that of Ca^2+^ (3.291 nm), which confirms less flexibility (stiffer backbone) of LPSG molecular chains in the solution containing divalent cations. The *q* values acquired in the salt solutions for LPSG were greater than those obtained for sage seed gum (Yousefi et al., 2014) and *Descurainia Sophia* seed gum (Sherahi et al., 2018), but were lower than those of xanthan (Camesano & Wilkinson, 2001).

### 3.9 Molecular conformation

Base on the study of Morris et al. (1981), when the slop of logarithm of *η*_*sp*_ versus logarithm of *C*[*η*], so‐called master curve, is close to 1.4, it implies the dilute solution regime in which there is no coil entanglement or coil overlaps, whilst the slope close to 3.3 is corresponding to the concentrated regime. It was found that the slopes of master curves for all the LPSG solutions were in the range of 1.02–1.22, reflecting no physical coil entanglement or coil overlapping in the dilute regime (Table 2). This issue was again proved where the value of Berry number (*C*[*η*]) for LPSG found to be lower than unity (0.10–0.79) at all conditions examined (Table 2). The exponent *b*, which is the slope of power‐law model (Equation (9)), was obtained at various solution conditions and it is represented in Table 2. It has been reported that in aqueous solution, *b* values greater than 1 are associated with the random coil conformation, while less values are related to the rod‐like conformation (Morris et al., 1981). This value for LPSG was 1.08–1.12 at the selected temperatures (25–65°C), and was 1.07–1.14 at the selected pH, and also was in the range of 1.14–1.28 and 1.06–1.17 in the presence of the ions and sugars, respectively. Accordingly, the molecular conformation of LPSG is probably random coil in all the selected solution conditions. The higher values obtained in the presence of ions may be as a result of shielding influence of charges on polyelectrolyte chains (Goycoolea et al., 1995). The values of *b* parameter increased when ions concentration increased, which exhibited that they are able to booster the random coil conformation of LPSG. Similar results have been reported for xanthan gum (Higiro et al., 2007), cress seed gum (Behrouzian et al., 2014), *Descurainia sophia* seed gum (Sherahi et al., 2018).

### 3.10 Shape function and swollen specific volume

The results obtained for the shape function (*υ*) and swollen specific volume (*υ*_*s*_) of LPSG macromolecules at the various solution conditions are depicted in Table 5. It was observed that every 20°C increment in temperature resulted in 20% and 19% decrement in *υ*_*s*_, indicating the diminution of the coil dimension of LPSG macromolecules or the solvent power (Yousefi et al., 2014). According to the description for quantitative values of coil shape function provided by Antoniou et al. (2010), the LPSG molecules have oblate or prolate coil shape at the selected temperature. As it is evident from the results, the value of *υ* increased up to 0.99 with increasing temperature, demonstrating more contraction at higher temperature. Similar results have been reported for dextran (Antoniou et al., 2010) and Balangu seed gum (Amini & Razavi, 2012). A greater reduction in the *υ*_*s*_ value of LPSG as a result of more molecular contraction was occurred in the presence of Ca^2+^ (87%–98%) compared to Na^+^ (72%–92%), which was in accordance with the results of intrinsic viscosity (Table 2). The shape function of LPSG molecules in the presence of the ions was roughly found to be ellipsoidal shape. Based on the statement of Montesi et al. (2004), this ellipsoidal shape reveals that the monomers of polymers effectively attract each other to minimize their contacts with the solvent molecules; therefore, a roughly spherical or ellipsoidal shape forms which have lesser flexibility. In the presence of sucrose and lactose, the *υ*_*s*_ values of LPSG decreased (38%–81%), and this decrement was more evident at higher concentrations of the sugars. This result was in agreement with the results attained for some emerging carbohydrate polymers (Amini & Razavi, 2012; Fathi et al., 2017; Mirabolhassani et al., 2016). As it can be seen (Table 5), the shape factor in the sugar solutions of LPSG is oblate or prolate; however, at higher concentrations the coil shape tends to be spherical. A decrease in value of *υ*_*s*_ up to 47% was observed, when the pH of solutions decreased from 7 to 3, and the shape factor in this range of pH was oblate or prolate shape for LPSG coils (Antoniou et al., 2010).

**TABLE 5:** The values of shape factor, swollen specific volume, coil radius and volume of LPSG estimated by the equations (10–13) at the selected solution conditions^ab^

Solution conditions		υ (−)	υ_s_ (dl/g)	*R*_*Coil*_ (nm)	*V*_*Coil*_ (nm^3^)
Temperature (°C)	25	0.78 ± 0.00^e^	17.33 ± 0.08^a^	1.71 ± 0.03^a^	20.93 ± 0.72^a^
	35	0.82 ± 0.03^d^	15.86 ± 0.11^b^	1.68 ± 0.02^ab^	19.85 ± 0.53^b^
	45	0.89 ± 0.01^c^	13.54 ± 0.05^c^	1.64 ± 0.02^b^	18.46 ± 0.55^c^
	55	0.93 ± 0.00^b^	12.45 ± 0.09^d^	1.62 ± 0.01^bc^	17.79 ± 0.20^d^
	65	0.99 ± 0.03^a^	10.96 ± 0.06^e^	1.59 ± 0.01^c^	16.82 ± 0.28^e^
NaCl (mM)	10	2.02 ± 0.08^d^	3.67 ± 0.06^a^	1.40 ± 0.01^a^	11.49 ± 0.18^a^
	50	2.19 ± 0.03^c^	2.37 ± 0.04^b^	1.24 ± 0.01^b^	7.98 ± 0.18^b^
	100	2.49 ± 0.11^b^	1.85 ± 0.08^c^	1.19 ± 0.03^c^	7.06 ± 0.27^c^
	200	2.96 ± 0.16^a^	1.31 ± 0.03^d^	1.13 ± 0.01^d^	6.04 ± 0.13^d^
CaCl_2_ (mM)	10	2.12 ± 0.15^d^	2.51 ± 0.13^a^	1.26 ± 0.01^a^	8.37 ± 0.16^a^
	50	3.33 ± 0.19^c^	1.04 ± 0.04^b^	1.09 ± 0.02^b^	5.42 ± 0.22^b^
	100	4.40 ± 0.33^b^	0.60 ± 0.02^c^	0.99 ± 0.01^c^	4.06 ± 0.09^c^
	200	5.55 ± 0.20^a^	0.38 ± 0.02^d^	0.92 ± 0.02^d^	3.26 ± 0.11^d^
Sucrose (%)	10	1.08 ± 0.06^c^	9.32 ± 0.10^a^	1.55 ± 0.02^a^	15.59 ± 0.44^a^
	20	1.27 ± 0.10^b^	6.84 ± 0.17^b^	1.48 ± 0.02^b^	13.57 ± 0.46^b^
	30	1.86 ± 0.09^a^	3.63 ± 0.08^c^	1.36 ± 0.02^c^	10.53 ± 0.29^c^
	40	1.94 ± 0.12^a^	3.37 ± 0.11^d^	1.34 ± 0.00^c^	10.07 ± 0.07^d^
Lactose (%)	2.5	1.11 ± 0.03^d^	10.72 ± 0.21^a^	1.64 ± 0.02^a^	18.47 ± 0.60^a^
	5	1.24 ± 0.05^c^	8.02 ± 0.16^b^	1.54 ± 0.01^b^	15.29 ± 0.40^b^
	7.5	1.63 ± 0.14^b^	5.26 ± 0.15^c^	1.47 ± 0.02^c^	13.30 ± 0.42^c^
	10	1.88 ± 0.07^a^	4.26 ± 0.07^d^	1.44 ± 0.01^d^	12.50 ± 0.24^d^
pH	3	1.09 ± 0.02^a^	10.26 ± 0.11^c^	1.61 ± 0.02^c^	17.39 ± 0.53^c^
	5	0.84 ± 0.00^b^	15.54 ± 0.07^b^	1.69 ± 0.02^ab^	20.28 ± 0.31^ab^
	7	0.71 ± 0.00^c^	19.49 ± 0.13^a^	1.73 ± 0.03^a^	21.51 ± 0.76^a^

Abbreviation: LPSG, *Lepidium perfoliatum* seed gum.

^a–d^ For each solution condition, values in columns with different letters are significantly different (*p* < .05).

### 3.11 Coil radius and volume

Based on the value of the *M*_*w*_ reported aforementioned (2.34 × 10^5^ g/mol, at 25°C), the coil radios (*R*_*coil*_) and the corresponding volume (*V*_*coil*_) of LPSG macromolecules were determined as a function of the solution conditions (Table 5). The ion type and the ionic concentration revealed significant reduction influences on the values of *R*_*coil*_ and *V*_*coil*_. Regardless of the ion type, both of the coil parameters were reduced to a greater extent in divalent solutions and higher concentrations. As expected, these results were in agreement with the results reported for intrinsic viscosity (Table 2), demonstrating these parameters are profoundly affected by the hydrodynamic volume of macromolecules in solutions. So, the higher the ions concentration, the more extent of the molecular contraction and the lower the values of *R*_*coil*_ and *V*_*coil*_ (Fathi et al., 2017; Yousefi et al., 2014). Comparatively, the estimated value of 1.71 nm for *R*_*coil*_ of LPSG and the corresponding value of 20.93 nm^3^ obtained for *V*_*coil*_ parameter (in deionized water, 25°C) were approximately close to the value of 1.46 nm and 13.03 nm^3^ stated for basil seed gum (Mirabolhassani et al., 2016), but were lesser than those reported for other Carbohydrate Polymers (Amini & Razavi, 2012; Fathi et al., 2016; Fathi et al., 2017; Hesarinejad et al., 2015; Yousefi et al., 2014).

Both of the coil parameters were decreased in the presence of selected sugars particularly at higher concentration. Comparatively, it is evident that the reduction impact of lactose on the coil dimensions parameters of LPSG molecules is more prominent. This difference may be influenced by the difference of the molecular conformation and spatial orientation of the disaccharide molecules. In this case, similar results have been reported for Balangu seed gum (Amini & Razavi, 2012), basil seed gum (Mirabolhassani et al., 2016), and *Prunus armeniaca* gum (Fathi et al., 2017). The coil parameters were also diminished (7% for *R*_*coil*_ and 20% for *V*_*coil*_) as the pH of solutions was decreased from 7 to 3, indicating a reduction in the size of LPSG coils in the acidic pH, that it is reported to be influenced by the decrease in molecular weight of macromolecules (Bak & Yoo, 2018).

## 4 CONCLUSION

A little difference between the compositions of LPSG powder, which may affect its properties in the solutions, can be considered as a limitation of this study. In this work, the average molecular weight of LPSG (*M*_*w*_) was found to be 2.34 × 10^5^ g/mol, at 25°C, which indicates this carbohydrate polymer has low molecular weight. The Higiro model (Equation (4)) represented better fitting results for estimation of the intrinsic viscosity of LPSG at the selected conditions. In comparison, the intrinsic viscosity of LPSG in deionized water was relatively lower than that for most of commercial hydrocolloids. The reduction sensitivity of intrinsic viscosity of LPSG in the presence of Ca^2+^, lactose and acidic pH was found to higher than the other conditions. The results revealed that LPSG had exactly low level of flexibility (553.08 K). The anionic nature of LPSG was identified at a broad range of pH (3–11). A stiffer chain was obtained for LPSG in the presence of divalent cations (Ca^2+^) due to a greater extent of contraction. Addition of LPSG to water (in dilute solution domain), to some extent, diminished the surface activity of water. Based the value of *b* component, the molecular conformation of LPSG was random coil in all the selected solution conditions. At higher solution temperature and in the presence of higher concentrations of ions and sugars, more contraction (lower values of *R*_*coil*_, *V*_*coil*_ and *υ*_*s*_) in molecular chains of LPSG was observed. In conclusion, it can be stated that the solvent quality significantly decreases as the concentration of cosolutes exist in solution and/or the temperature raises. Surprisingly, according to the findings of this research, lower degree of flexibility obtained for LPSG molecules makes it an appropriate candidate to be applied as encapsulator in food and pharmaceutical systems. This issue will be taken into consideration in subsequent researches.

## CONFLICT OF INTEREST

The authors declare that they have no conflict of interest.

## Data Availability

The data that support the findings of this study are available from the corresponding author upon reasonable request.
